# Who is really shaping global health reform?

**DOI:** 10.1371/journal.pgph.0007342

**Published:** 2026-09-18

**Authors:** Filipa Alpeza, Afifah Rahman-Shepherd

**Affiliations:** 1 Stockholm School of Economics, Stockholm, Sweden; 2 Saw Swee Hock School of Public Health, National University of Singapore, Singapore; PLOS: Public Library of Science,UNITED STATES OF AMERICA

The global health system is under unprecedented pressure to reform. Reform discussions have centred on advancing health sovereignty, with countries that have long depended on external assistance committing to take over the responsibility for their own health systems. A major component of the reform agenda has thus become elevating country leadership while restricting the mandates of, and, in some instances, merging or closing, global health organisations (GHOs) [1]. While these organisations have contributed to significant health gains, they have also perpetuated power imbalances between the Global North and the Global South, and many no longer reflect the priorities and growing capacities of the countries they were intended to serve.

Despite the spotlight on organisational reform, reform proposals have seldom specified which among the hundreds of GHOs should undergo change, how, and by whom [2]. Such ambiguity impedes action yet seems to persist across reform debates and processes. Part of the issue, we believe, is a tension in how GHOs are understood, perceived, and treated within contemporary global health discourse.

A state-centric view might treat international organisations as arenas where states exert their political, financial, and technical influence. In this view, GHOs are often reduced to objects of reform, and reform is framed as a matter of political will, agendas within states, and the distribution of power between them. Obedience from GHOs is assumed, provided states reach agreement and issue clear directives [3]. However, this overlooks the organisations themselves: who leads and staffs them, the organisational logic that shapes their motives, and whether they have an incentive to radically depart from status quo. A less state-centric view might instead treat international organisations as actors with characters and interests of their own, more complex than obedient agents awaiting principal instruction [4]. This way of thinking prompts us to pay more attention to the internal dynamics and organisational behaviours that give GHOs ‘actorness’, in other words, their own capability to act purposively in international affairs, including in shaping global health reform [5,6].

**Fig 1:**
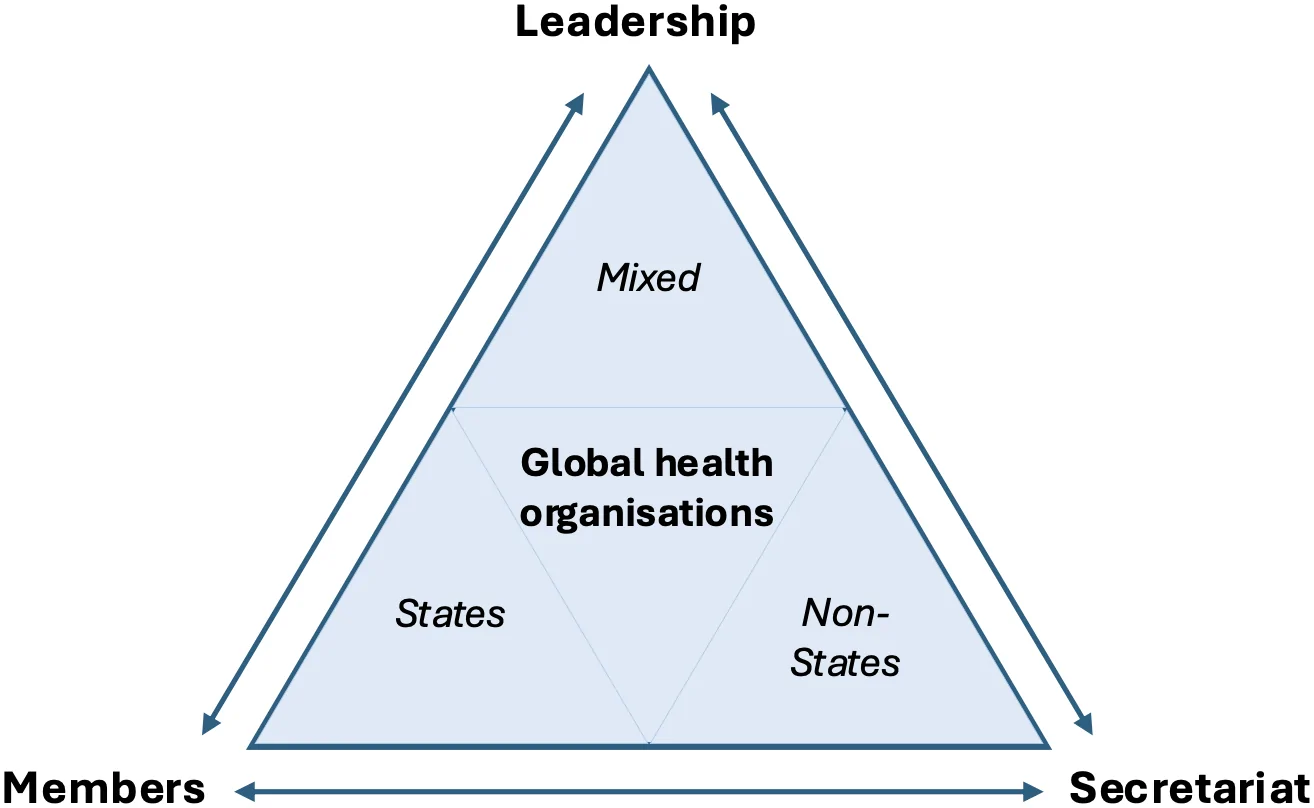
A conceptual framework for understanding the nature of GHOs.

We conceptualise GHOs in Fig 1 using three broad archetypes—state-based membership (states only), independent membership (non-state only), and mixed membership (state and non-state)—and the three key players that comprise most international organisations—leader(ship), secretariat, and members [3]. Conceptualising GHOs in this way allows us to consider how the position of GHOs in reform might be more actively negotiated from within.

The single most important state-based membership GHO, the WHO, is playing a critical role in global health reform. In December 2025, the Director General of WHO proposed hosting a joint process to support reforms of the global health architecture. Having secured approval from the Executive Board, the Director General tasked the WHO Secretariat with designing the process. The Secretariat, in many cases, has this ability to shape internal operations and execute organisational agendas [3]. While the WHO is a state-based membership GHO, the joint process will be led by a Taskforce of up to 25 members, of which 11 are reserved for representatives beyond WHO Member States, including five of the largest mixed membership GHOs: Gavi, Global Fund, Coalition for Epidemic Preparedness Innovations, Unitaid, and the Pandemic Fund. The entanglement of state-based and mixed membership GHOs in a critical reform process reflects the extent to which network power currently characterises and shapes global governance for health [6–8]. Mixed membership GHOs hold seats in around a third of all mixed membership GHOs. Gavi and the Global Fund, for instance, have governing seats in five and four others respectively [8]. The rise of ‘multi-stakeholderism’ in global health has been described as a ‘Trojan horse’ for dominant actors, state and non-state alike, to exert more control over different types of GHOs, maintaining their positions of power and influence in the global health system, including its reform [7].

Gavi, alongside other mixed membership GHOs, is also playing an increasingly active and visible role in reforms broadly. Since 2024, the Chief Executive Officer of Gavi has authored several articles in The Lancet Global Health announcing the ‘Gavi Leap’ and articulating the vision, mandate, and future of the organisation in global health reform, including which functions Gavi should retain [9]. According to the Gavi Leap report, the Secretariat has already begun implementing extensive changes to the organisation’s governance, management, and financing structures. Similarly, the former Executive Director of Unitaid published an op-ed in The Council on Foreign Relations on global health reform, framing the Unitaid model as ‘among the most cost-effective in global health’ [10]. As geopolitical tensions rise and financing for global health rapidly recedes, dynamics between states and GHOs are increasingly tested. For instance, leadership within the Global Fund negotiated a waiver from the United States foreign assistance policy that prohibits funding for programs supporting abortion, diversity, equity, and inclusion, and gender ‘ideology’. The negotiations demonstrate the ability of GHOs to confront their members, even their biggest funders, and successfully advocate their own agendas [11]. Given Gavi and the Global Fund are GHOs at the forefront of reform conversations, how their leader(ship) navigate and manage their members is key to unveiling the potential tensions, interests, and resourcing of GHOs as they enter into various reform processes.

Despite global civil society and non-government organisations accounting for the vast majority of global health actors, independent membership GHOs remain the least involved—at least, formally—in global health reform [12–14]. The WHO-hosted reform process, for instance, has been widely criticised for excluding representation from civil society and communities in the proposed Taskforce [12,15]. Regardless of their involvement (or lack thereof), independent GHOs have been nonetheless vocal about reform. When the UN Secretary General proposed sunsetting UNAIDS by the end of 2025, there was widespread disproval from civil society and non-government organisations. The Health Architecture Reimagined Civil Society Organisations launched their Consortium in 2025, convening nearly 140 non-state members to develop their own proposals for reform. The NCD Alliance, for example, has been urging stronger prioritisation of NCDs in the global health system, echoing those with lived experience of NCDs and other chronic conditions who do not see themselves nor their communities reflected in the current reform agenda [12].

Behind the shifting sands of global health today, there are GHOs with agency and authority, which they exercise in ways that are consequential for global health reform. While states may provide the political steer for reform, change greatly depends on the buy-in of organisations responsible for implementing it, and a common understanding among the leadership, secretariats, and members of GHOs. We therefore urge the proponents of global health reform to pay much greater attention to the actorness of GHOs and their internal logic, and the GHOs themselves to transparently declare their positions and interests in various reform processes. Finally, we call for the creation of robust accountability mechanisms for delivering on global health reform that operate independently of the organisations affected [15]. Without recognising GHOs as agentic, there is a risk that reform efforts overlook key actors and interests at play, affecting the likelihood of any proposed changes materializing.
